# Investigation of the Formation Process of Two Piracetam Cocrystals during Grinding

**DOI:** 10.3390/pharmaceutics3040706

**Published:** 2011-10-12

**Authors:** Sönke Rehder, Marten Klukkert, Korbinian A. M. Löbmann, Clare J. Strachan, Albrecht Sakmann, Keith Gordon, Thomas Rades, Claudia S. Leopold

**Affiliations:** 1 Department of Chemistry, Division of Pharmaceutical Technology, University of Hamburg, Bundesstraße 45, Hamburg 20146, Germany; 2 School of Pharmacy, University of Otago, 18 Frederick Street, Dunedin 9054, New Zealand; 3 Department of Chemistry, MacDiarmid Institute of Advanced Materials and Nanotechnology, University of Otago, Union Place West, Dunedin, New Zealand

**Keywords:** piracetam, cocrystal, chemometrics, formation kinetics, grinding

## Abstract

Cocrystal formation rates during dry grinding and liquid-assisted grinding were investigated by X-ray powder diffractometry and Raman spectroscopy. Two polymorphic forms of piracetam were used to prepare known piracetam cocrystals as model substances, *i.e.*, piracetam-citric acid and piracetam-tartaric acid cocrystals. Raman spectroscopy in combination with principal component analysis was used to visualize the cocrystal formation pathways. During dry grinding, cocrystal formation appeared to progress via an amorphous intermediate stage, which was more evident for the piracetam-citric acid than for the piracetam-tartaric acid cocrystal. It was shown that liquid-assisted grinding led to faster cocrystal formation than dry grinding, which may be explained by the higher transformation rate due to the presence of liquid. The cocrystal formation rate did not depend on the applied polymorphic form of the piracetam and no polymorphic cocrystals were obtained.

## 1. Introduction

Pharmaceutical cocrystals can be defined as stoichiometric multiple component substances formed by active pharmaceutical ingredients (API) and cocrystal formers. At least two components of a cocrystal must be solid under ambient conditions [1].

Cocrystals are gaining increasing interest in the pharmaceutical community, because they differ in their physicochemical properties from single-component crystals, e.g., melting point [2], hydration stability [3], UV light stability [4], hygroscopic properties [5], dissolution behavior [6], and bioavailability [7].

Although most cocrystals have been found by chance, an increased understanding of the cocrystal formation process during the last few decades has led to more systematic cocrystal design. Two approaches are common practice: one is based on a structural fit of the compounds, *i.e.*, similarities in molecule packing, and the other is based on specific pair wise interactions, so-called supramolecular synthons [8]. The API and cocrystal former interact via non-ionic and non-covalent intermolecular interactions, such as van der Waals forces, π-π-interactions, and most importantly, hydrogen bonding. Hence, the presence of free hydrogen bond donors and acceptors is usually a prerequisite for cocrystal formation [2]. Supramolecular assemblies of cocrystals may be based on homosynthons, such as acid-acid interactions, and heterosynthons, for example acid–amide interactions [1].

**Figure 1.**
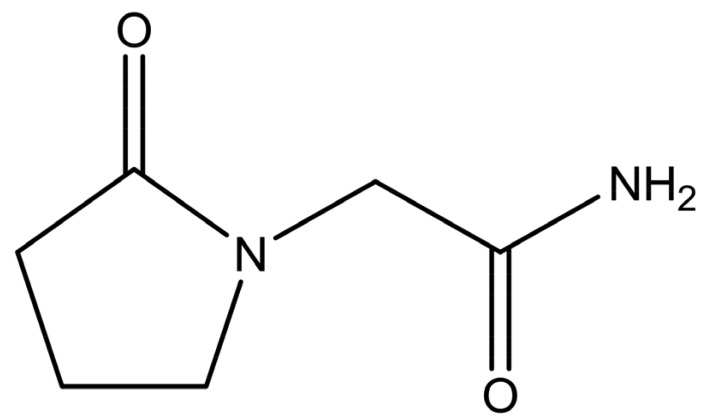
Chemical structure of piracetam.

Piracetam (2-oxo-1-pyrrolidineacetamide, shown in Figure 1) is a nootropic substance, used for the treatment of memory and balance problems. Piracetam is a neutral molecule, containing two different amide moieties, which could form heterosynthons with carboxylic acid or hydroxyl groups. Five anhydrous polymorphic forms (forms I–V) and two hydratesof this drug have been reported [9,10]. Form III was found to be the thermodynamically stable polymorph at ambient conditions [11]. The presence of different polymorphic forms increases the chance for cocrystal formation because polymorphism is based on molecular flexibility. Hence, it may be easier to pack such a molecule in a different crystal lattice arrangement with another substance than is the case for structurally more rigid molecules [12].

As a result of the ability to form heterosynthons on the one hand and structural flexibility on the other hand, piracetam is a suitable model substance for the investigation of cocrystal formation. It is thus not surprising that several piracetam cocrystals are described in the literature. In 2005, Vishweshwar *et al.* characterized piracetam cocrystals formed with 2,5-dihydroxybenzoic acid (Cambridge Structural Database (CSD) reference code: DAVPAS) and 4-hydroxybenzoic acid (CSD reference code: DAVPEW) by slow evaporation of acetonitrile, slurrying in water, and dry-grinding using Fourier transformation infrared spectroscopy (FTIR), differential scanning calorimetry (DSC), X-ray powder diffractometry (XRPD), and single-crystal X-ray diffractometry (SC-XRD) [13]. Liao *et al.* examined the formation of piracetam cocrystals with different isomers of dihydroxybenzoic acid by crystallization from acetonitrile and characterized the cocrystals by DSC, FTIR, and XRPD [14]. Recently, Viertelhaus *et al.* described a screening experiment for piracetam cocrystals, using Raman microscopy, FT-Raman spectroscopy, XRPD, SC-XRD, dynamic vapor sorption, thermogravimetry coupled with FTIR, and DSC as characterization techniques. Piracetam cocrystals with *L*-(+) tartaric acid (*L*-(+)-2,3-dihydroxybutanedioic acid) (CSD reference code: RUCDUP), racemic 2-hydroxy-2-phenylacetic acid (CSD reference code: RUCFIF), *L*-2-hydroxy-2-phenylacetic acid (CSD reference code: XOZSOV), and citric acid (2-hydroxypropane-1,2,3-tricarboxylic acid) at molar ratios of 1:1 (CSD reference code: RUCFAX) and 3:2 (CSD reference code: RUCFEB) as well as an ethanol solvate of the piracetam-2-hydroxypropane-1,2,3-tricarboxylic acid cocrystal (not published in the CSD) were detected. These cocrystals were prepared by solvent evaporation, solution crystallization, and liquid-assisted grinding [5].

Cogrinding of an API and a cocrystal former is an important technique for cocrystal preparation and especially for cocrystal screening [5,15–17]. Three mechanisms are discussed for cocrystal formation by dry-grinding: molecular diffusion, intermediate formation of eutectic mixtures, and intermediate formation of an amorphous phase. Usually a more effective grinding method is liquid-assisted grinding [5,9,18], although the mechanism, and especially the role of the liquid, is not yet fully understood. Some authors suggest that a small amount of liquid may act as a lubricant for the reaction, while others state that the liquid provides a medium to enhance molecular diffusion [19]. While many research articles have been published regarding cocrystal characterization [5], screening [20], design [21], and storage stability [15], little work has been done to understand the kinetics of cocrystal formation during grinding. Chieng *et al.* followed the cocrystallization process of different solid-state forms of carbamazepine during dry-grinding with nicotine amide by combining XRPD with a multivariate data analysis approach [15], concluding that cocrystal formation using carbamazepine hydrate is faster than using the meta-stable polymorphic form I of the drug, which in turn was faster than using the stable polymorphic form III. Principal component analysis (PCA) provided a valuable tool to visualize the cocrystal formation process.

The aim of this study was to gain a deeper insight into the formation of two known piracetam cocrystals, *i.e.* piracetam-citric acid and piracetam-tartaric acid, during grinding. In the first part of the study cocrystal formation was investigated as a function of the grinding technique and the polymorphic form of the API, using XRPD, DSC, and Raman spectroscopy. In the second part PCA of the Raman spectra was performed to provide a more detailed insight into the cocrystallization mechanism.

## 2. Experimental Section

### 2.1. Materials

Piracetam (M_W_: 142.16 g/mol) was purchased from Hangzhou Dayangchem, China. Purity was confirmed by high performance liquid chromatography. The polymorphic form was determined to be form III by XRPD and DSC. However, to exclude impurities of other piracetam polymorphs, form III was used after recrystallization from methanol at ambient conditions. Piracetam form I was obtained by heating form III at 160 °C for 5 min and subsequent cooling to room temperature. Citric acid (M_W_: 192.13 g/mol) was purchased from AppliChem, Germany, and *L*-(+)-tartaric acid (M_W_: 150.09 g/mol) was purchased from Ajax Chemicals, Australia; both compounds were of pharmaceutical grade and were used as received.

### 2.2. Methods

#### 2.2.1. Physical mixing

The cocrystal formers were ground before mixing to achieve the same particle size as the API. Physical mixtures were obtained by gently mixing API and cocrystal formers at a 1:1 molar ratio in a glass mortar with a glass pestle for 1 min.

#### 2.2.2. Grinding

##### 2.2.2.1. Dry-grinding

Dry-grinding was performed by co-milling piracetam with citric acid and tartaric acid, respectively, at a 1:1 molar ratio in 25 mL stainless steel milling jars using an oscillatory ball mill (Retsch MM301, Germany). Each jar contained three 9 mm stainless steel balls. Milling was carried out for predefined time periods from 1 min to 30 min at a frequency of 30 Hz.

##### 2.2.2.2. Liquid-assisted grinding

For liquid-assisted grinding, the same process parameters as for dry-grinding were used. Additionally, 16.6 μL of water and 166 μL of ethyl acetate were added to prepare the piracetam-citric acid cocrystal before the milling process was started. For piracetam-tartaric acid cocrystal preparation, 16.6 μL of water were added [5].

#### 2.2.3. Characterization methods

##### 2.2.3.1. X-ray powder diffractometry (XRPD)

Differences in crystal lattice configuration were examined using a PANalytical X'Pert PROMD diffractometer (PW3040/60, Philips, The Netherlands), with CuKα radiation at a wavelength of 1.54 Å in continuous scanning mode. The step size was 0.0084 °2θ and the scanning rate was 0.1285 °2θ/min. Powder samples were analyzed in aluminium sample holders and scanned at 40 kV and 30 mA from 5 to 35 °2θ. The powder diffraction patterns were analyzed with X'Pert Highscore software (version 2.2.0) and plotted with OriginPro 7.5. The theoretical cocrystal patterns were calculated on the basis of the Cambridge Structural Data base (CSD 5.32, November 2010) [22] using ConQuest 1.13 [23] by Mercury software CSD 2.4 [24] (Cambridge Crystallographic Data Centre, UK).

##### 2.2.3.2. Differential scanning calorimetry (DSC)

To confirm the XRPD results, DSC was performed. Each sample was analyzed in triplicate. The material was weighed (1–5 mg) into a TA instruments standard aluminium pan using a micro balance and tweezers. The pan was covered with a lid and crimped using a TA crimper. The reference pan was crimped similarly to the sample pans but without any substance.

Thermograms were recorded on a Q100 V8.2 Build 268, (TA Instruments, USA) under a constant nitrogen gas flow of 50 mL/min. The DSC apparatus was calibrated with regard to temperature and enthalpy using indium as a standard. The heating rate was set to 10 K/min in a range from 20 to 180 °C. To determine any thermal events the TA Universal Analysis 2000 software (version 4.0c) was used.

##### 2.2.3.3. FT-Raman spectroscopy

FT-Raman spectra were recorded using a Bruker FRA 106/S FT-Raman spectrometer (Bruker, Germany), equipped with a Coherent Compass 1064-500N laser (Coherent, USA), attached to a Bruker IFS 55 FT-IR interferometer, and a D 425 Ge diode detector. The laser wavelength was 1064 nm and laser power 120 mW. To monitor the wave number accuracy sulfur was used as a reference standard. Measurements were performed in triplicate (each spectrum was averaged over 64 scans) at a resolution of 4 cm^−1^. Spectra were displayed using the OPUS 5.0 software.

##### 2.2.3.4. Chemometrics

Spectral changes due to cocrystal formation were visualized by performing principal component analysis (PCA) of the Raman spectra. The data were pre-treated with a standard normal variate algorithm and scaled by mean centering. Multivariate data analysis was performed with The Unscrambler X (version 10, Camo, Norway). The spectral regions between 1800 cm^−1^ and 2700 cm^−1^ and above 3100 cm^−1^ were excluded.

## 3. Results and Discussion

**Figure 2.**
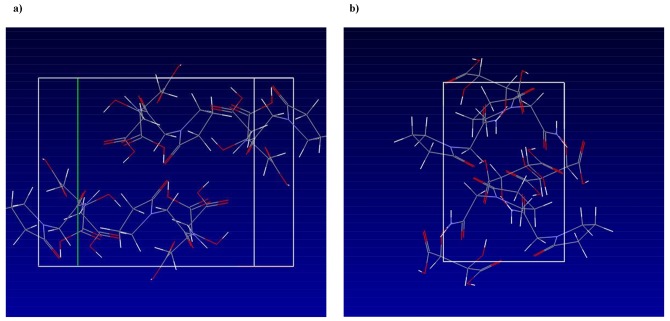
(**a**) 3D structure of the piracetam-citric acid unit cell. (**b**) 3D structure of the piracetam-tartaric acid unit cell (Cambridge Structural Database 2011) [22,23].

The substances investigated in this study were characterized by XRPD, DSC, and Raman spectroscopy. The piracetam-citric acid and piracetam-tartaric acid cocrystal structure was thoroughly described by Viertelhaus *et al.* [5], a schematic overview over the interactions within the unit cells is presented in Figure 2 (Cambridge Structural Database 2011) [22,23].

**Figure 3.**
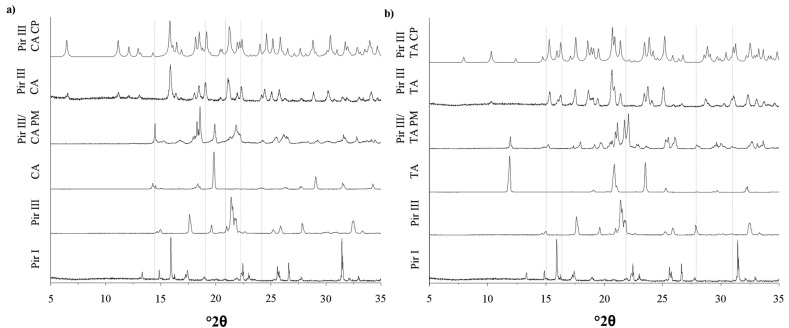
(**a**) XRPD patterns of piracetam form I (Pir I) and form III (Pir III), citric acid (CA), physical mixture of piracetam form III and citric acid (Pir III/CA PM), piracetam-citric acid cocrystal (Pir III CA), and calculated piracetam-citric acid cocrystal pattern (Pir III CA CP). (**b**) XRPD patterns of piracetam form I (Pir I) and form III (Pir III), tartaric acid (TA), physical mixture of piracetam form III and tartaric acid (Pir III/TA PM), piracetam-tartaric acid cocrystal (Pir III TA), and calculated piracetam-tartaric acid cocrystal pattern (Pir III TA CP).

**Figure 4.**
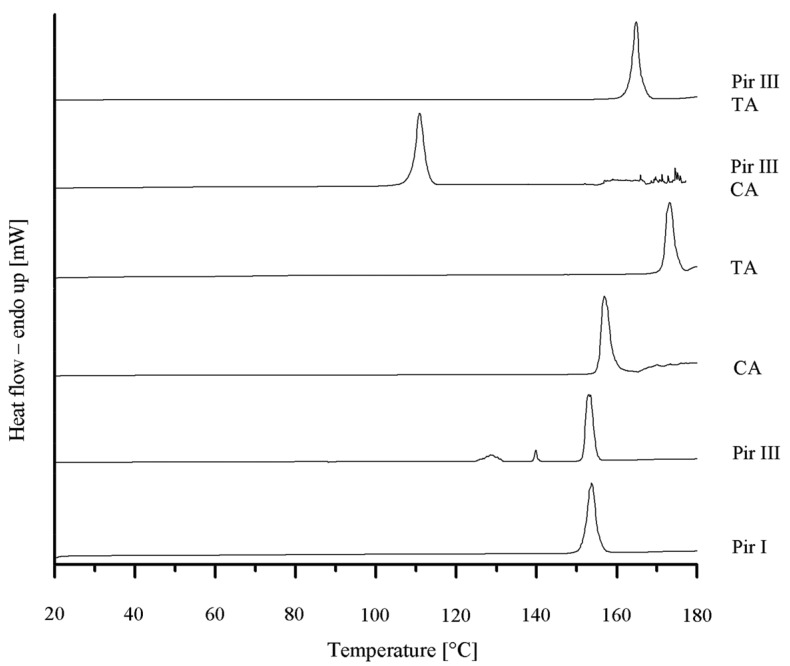
DSC thermograms of piracetam form I (Pir I) and form III (Pir III), citric acid (CA), tartaric acid (TA), piracetam-citric acid cocrystal(Pir III CA), and piracetam-tartaric acid cocrystal (Pir III TA).

**Figure 5.**
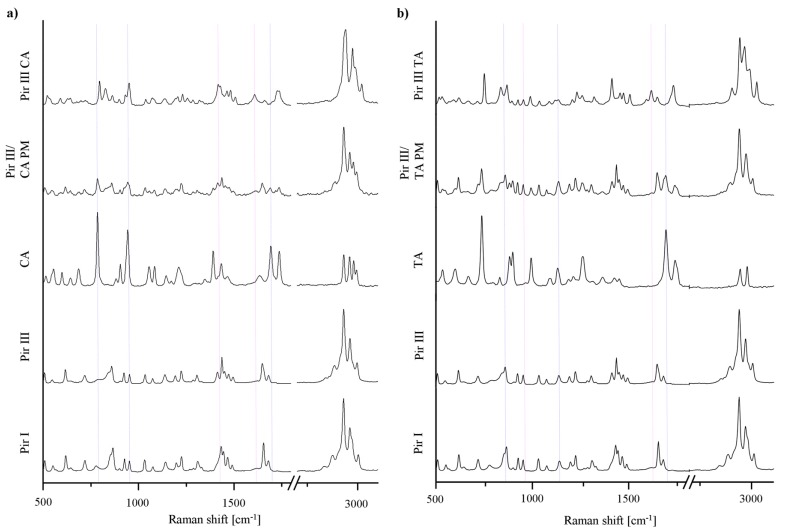
(**a**) Raman spectra of piracetam form I (Pir I) and form III (Pir III), citric acid (CA), physical mixture of piracetam form III and citric acid (Pir III/CA PM), and piracetam-citric acid cocrystal (Pir III CA). (**b**) Raman spectra of piracetam form I (Pir I) and form III (Pir III), tartaric acid (TA), physical mixture of piracetam form III and tartaric acid (Pir III/TA PM), and piracetam-tartaric acid cocrystal (Pir III TA).

The XRPD patterns, DSC thermograms, and the Raman spectra of the model API piracetam (polymorphic forms I and III), the cocrystal formers citric acid and tartaric acid, and the cocrystals are displayed in Figures 3–5. For clarity, only the data of the physical mixtures of piracetam form III and the cocrystal formers, rather than the individual components alone, are displayed. The calculated cocrystal XRPD patterns based on the single crystal data in the CSD are included in Figure 3. The characteristic peaks of the physical mixtures are highlighted by blue dotted lines. The patterns of the physical mixtures of piracetam form III and citric acid or tartaric acid, respectively, show combinations of the diffractograms of both compounds expressing API as well as cocrystal former peaks. In contrast, the cocrystal patterns are completely different to those of the physical mixtures, showing peaks which are not observable in the physical mixture patterns, because the crystal configuration differs significantly from the crystal lattices of the single components. The measured cocrystal patterns are in good agreement with the patterns calculated on the basis of the CSD (pink dotted lines).

To confirm the XRPD results, DSC was performed. In Figure 4 the various DSC thermograms are displayed. Piracetam form III shows three endothermic events at onset temperatures of 125 °C, 140 °C, and 150 °C. According to Maher *et al.* [25], the first endothermic event at 125 °C is the result of a partial transformation of form III into form I, which melts at 150 °C, while form III melts at 140 °C. Citric acid melts at 154 °C and L-tartaric acid at 170 °C. The melting onset temperature of the piracetam-citric acid cocrystal is 105 °C, while the piracetam–tartaric acid cocrystal melts at 160 °C. The melting points of the pure substances and the cocrystals are in good agreement with the values published in the literature [5,9,11,25].

Raman spectroscopy, which provides molecular level information, is a valuable technique for solid-state and cocrystal investigation [15,20].

In Figure 5 the characteristic Raman bands of the pure substances and the physical mixtures are highlighted by blue dotted lines; the cocrystal peaks are highlighted by pink dotted lines. The cocrystal spectra can easily be differentiated from the physical mixtures' spectra, since they show peaks which are not observed for the physical mixtures.

In the first part of the study the formation speeds of piracetam-citric acid cocrystals and piracetam-tartaric acid cocrystals were investigated as a function of different grinding techniques on the one hand and different polymorphic forms of piracetam on the other hand. Therefore, piracetam form I or form III were co-ground with citric acid and tartaric acid, respectively, by dry-grinding as well as by liquid-assisted grinding. The samples, milled for predefined time periods, were examined using XRPD and Raman spectroscopy.

**Figure 6.**
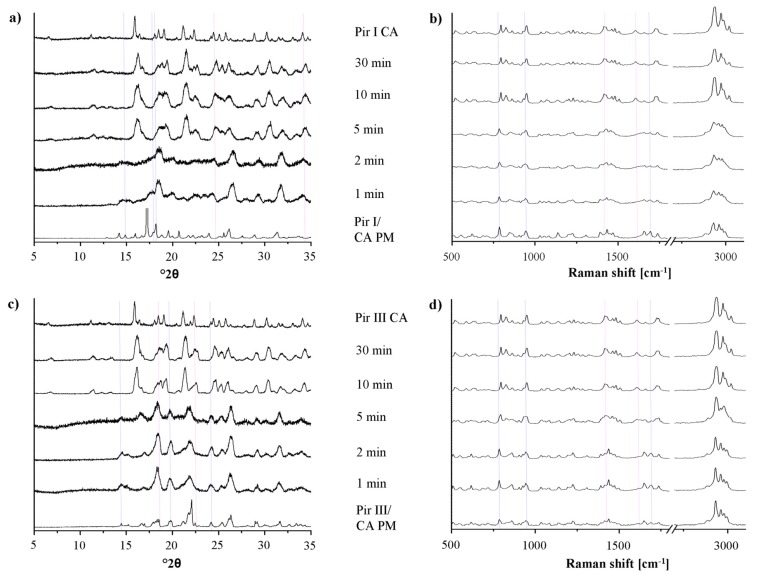
(**a**) XRPD patterns of the physical mixture of piracetam form I and citric acid (Pir I/CA PM), dry-ground for predefined time periods. The piracetam form I-citric acid cocrystal (Pir I CA) reference pattern is included. (**b**) Raman spectra of the physical mixture of piracetam form I and citric acid (Pir I/CA PM), dry-ground for predefined time periods. The piracetam form I-citric acid cocrystal (Pir I CA) reference spectrum is included. (**c**) XRPD patterns of the physical mixture of piracetam form III and citric acid (Pir III/CA PM), dry-ground for predefined time periods. The piracetam form III-citric acid cocrystal (Pir III CA) reference pattern is included. (**d**) Raman spectra of the physical mixture of piracetam form III and citric acid (Pir III/CA PM), dry-ground for predefined time periods. The piracetam form III-citric acid cocrystal (Pir III CA) reference spectrum is included.

In Figure 6, the XRPD patterns and the Raman spectra of the samples of piracetam form I and form III, dry-ground with citric acid, are shown. The patterns and spectra of the physical mixtures (blue dotted lines) and the cocrystal (pink dotted lines) are included as references.

Upon milling, significant changes in the XRPD patterns and in the Raman spectra can be detected. The intensity of the characteristic peaks of the physical mixture decreases, while that of the cocrystal peaks increases. After 10 min of milling, regardless of the piracetam polymorph used as starting material, the patterns and spectra only show characteristic cocrystal peaks, indicating complete cocrystal formation. Interestingly, the characteristic XRPD peaks and Raman bands of the cocrystals formed by bothpiracetam form I-citric acid and piracetam form III-citric acidmatch, indicating that the resulting cocrystals are identical.

For all samples, a loss of crystallinity of piracetam and citric acid during grinding is observed in the XRPD diffractograms and in the Raman spectra, identifiable by the broader peaks with lower intensity. This is known to occur during grinding [26]. Co-grinding at room temperature leads to partial amorphization, and further grinding can accelerate cocrystal formation. This cocrystal formation mechanism is typical for solids which are not volatile and which interact via hydrogen bonds. It has been suggested that cocrystal formation occurs via an intermediate amorphous stage of high energy and high molecular mobility [16]. Some samples were even completely amorphous after grinding, indicated by a broad halo in the XRPD pattern. These samples crystallized forming the cocrystal.

**Figure 7.**
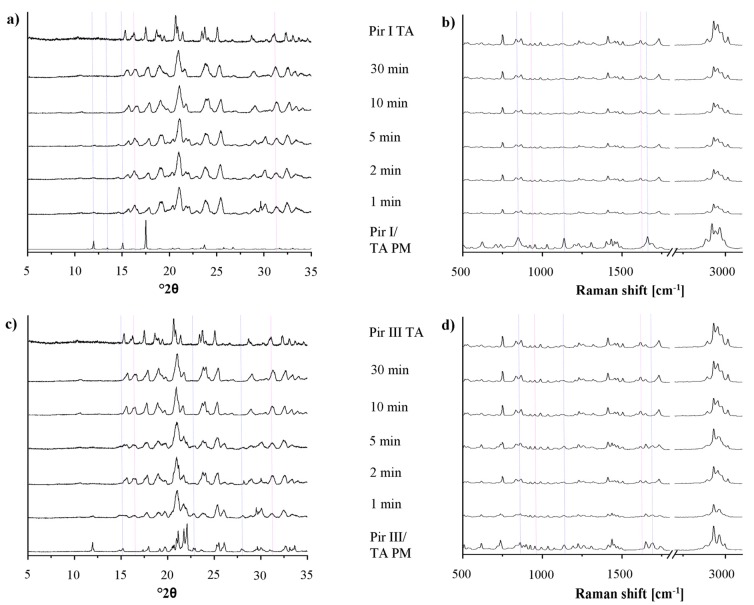
(**a**) XRPD patterns of the physical mixture of piracetam form I and tartaric acid (Pir I/TA PM), dry-ground for predefined time periods. The piracetam form I tartaric acid cocrystal (Pir I TA) reference pattern is included. (**b**) Raman spectra of the physical mixture of piracetam form I and tartaric acid (Pir I/TA PM), dry-ground for predefined time periods. The piracetam form I tartaric acid cocrystal (Pir I TA) reference spectrum is included. (**c**) XRPD patterns of the physical mixture of piracetam form III and tartaric acid (Pir III/TA PM), dry-ground for predefined time periods. The piracetam form III tartaric acid cocrystal (Pir III TA) reference pattern is included. (**d**) Raman spectra of the physical mixture of piracetam form III and tartaric acid (Pir III/TA PM), dry-ground for predefined time periods. The piracetam form III tartaric acid cocrystal (Pir III TA) reference spectrum is included.

In Figure 7, the XRPD patterns and the Raman spectra of piracetam form I and form III, dry-ground with tartaric acid at predefined milling times, are shown. Again, the intensity of the characteristic peaks of the physical mixture decreases, while the cocrystal peaks become more prominent with increasing milling times. Independent of the polymorphic form of piracetam, the physical mixture peaks disappear after 10 min of grinding and cocrystal formation is completed. This observation is surprising, as Chieng *et al.* showed that the thermodynamically less stable polymorph of carbamazepine forms cocrystals with nicotine amide faster during dry-grinding than the stable polymorph because of its higher energy [15]. This trend is not observed in the present study. Again, the cocrystals formed by piracetam form I and form III do not show polymorphism.

In the diffractograms, a loss of crystallinity, as it was shown for piracetam-citric acid cocrystals, cannot be observed with piracetam-tartaric acid. In addition, there was no evidence of amorphous material in the DSC thermograms (not shown) or Raman spectra detectable by the naked eye.

**Figure 8.**
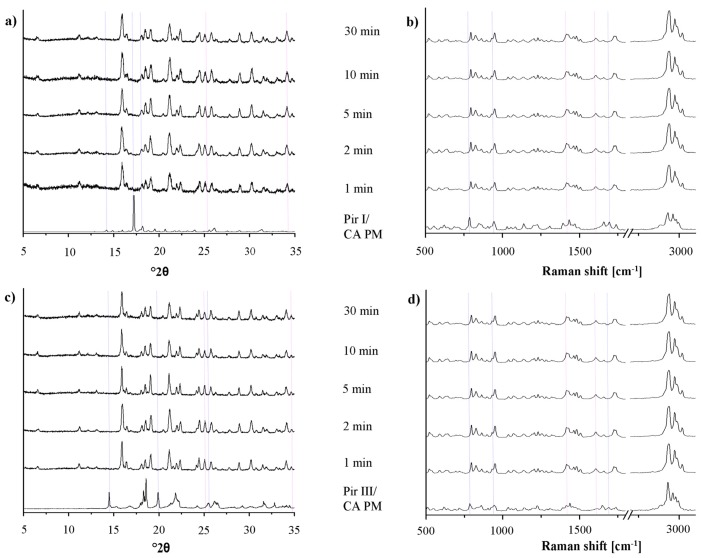
(**a**) XRPD patterns of the physical mixture of piracetam form I and citric acid (Pir I/CA PM), after liquid-assisted grinding for predefined time periods. (**b**) Raman spectra of the physical mixture of piracetam form I and citric acid (Pir I/CA PM), after liquid-assisted grinding for predefined time periods. (**c**) XRPD patterns of the physical mixture of piracetam form III and citric acid (Pir III/CA PM), after liquid-assisted grinding for predefined time periods. (**d**) Raman spectra of the physical mixture of piracetam form III and citric acid (Pir III/CA PM), after liquid-assisted grinding for predefined time periods.

**Figure 9.**
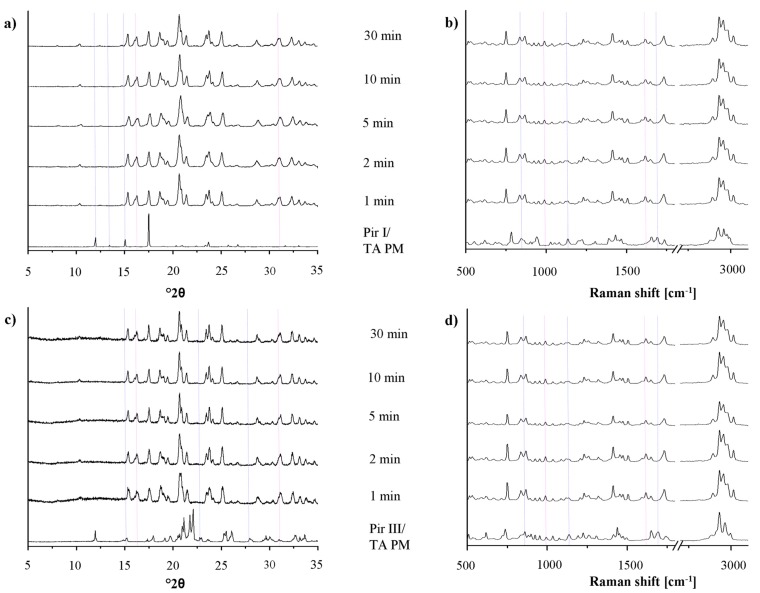
(**a**) XRPD patterns of the physical mixture of piracetam form I and tartaric acid (Pir I/TA PM), after liquid-assisted grinding for predefined time periods. (**b**) Raman spectra of the physical mixture of piracetam form I and tartaric acid (Pir I/TA PM), after liquid-assisted grinding for predefined time periods. (**c**) XRPD patterns of the physical mixture of piracetam form III and tartaric acid (Pir III/TA PM), after liquid-assisted grinding for predefined time periods. (**d**) Raman spectra of the physical mixture of piracetam form III and tartaric acid (Pir III/TA PM), after liquid-assisted grinding for predefined time periods.

Cocrystal formation during grinding may be enhanced by small amounts of liquid added to the milling jar before the milling process [18]. To compare the rate of cocrystal formation during this liquid-assisted grinding with dry-grinding, the XRPD patterns and the Raman spectra of piracetam form I or form III, co-ground with citric acid and tartaric acid, respectively, were recorded after the same milling times (Figures 8 and 9). Already after 1 min of milling, the patterns and spectra differ significantly from those of the physical mixture. Peaks characteristic of the physical mixture disappear from the XRPD patterns and the Raman spectra and only cocrystal peaks are detected. Obviously, cocrystal formation is already completed after 1 min of liquid-assisted grinding for both the piracetam-citric acid cocrystal and the piracetam-tartaric acid cocrystal.

On the one hand, the fast cocrystal formation may be explained by the higher molecular mobility of the API and the cocrystal formers as a result of their partial solubility in the liquids used in the experimental setup [18,19]. On the other hand, Viertelhaus *et al.* observed a loss of crystallinity in an equimolar mixture of piracetam and citric acid during liquid-assisted grinding [5]. This was not observed in the present study, probably because of the fast cocrystallization process.

As observed with the dry-grinding process, no differences are found between the cocrystal formation rates of the different polymorphic forms of piracetam during liquid-assisted grinding. According to the XRPD patterns, the cocrystals of piracetam and citric acid or tartaric acid obtained by liquid-assisted grinding and dry grinding, respectively, are identical. Raman spectroscopy (Figures 8b, 8d and 9b, 9d) and DSC (data not shown) support the XRPD findings.

In summary, it was found with both cocrystal systems that liquid-assisted grinding results in very fast cocrystal formation, which is completed within the first minute of grinding. Cocrystal formation during dry-grinding is a slower process and is completed only after 10 min. In the present study, the type of polymorphic form does not have a significant influence on the cocrystal formation rate, which is in contrast to findings published by Chieng *et al.* in 2009 for carbamazepine cocrystals [15]. Different polymorphic cocrystals are not obtained by dry-grinding or liquid-assisted grinding. Dry-grinding of piracetam andcitric acid leads to partial amorphization, which is suggested to be the cocrystallization mechanism. Amorphization is not obvious for formation of the piracetam-tartaric acid cocrystal.

**Figure 10.**
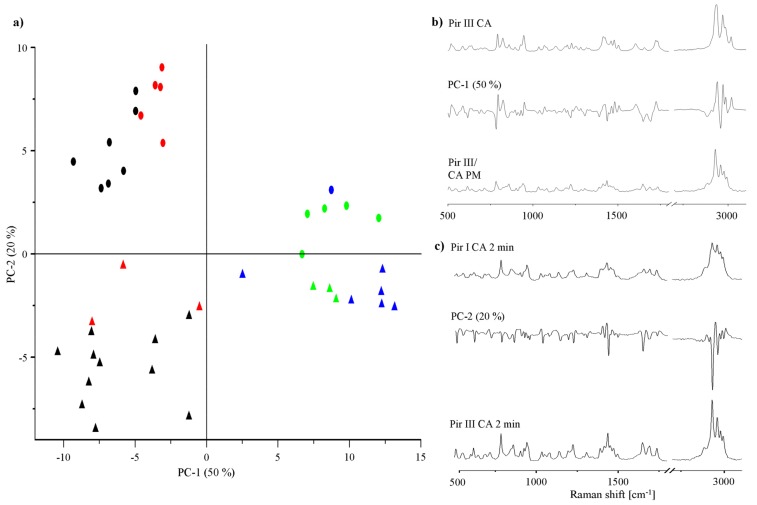
(**a**) PCA 2D score plot of piracetam form I (circles) or form III (triangles) dry-ground with citric acid. Black symbols represent non-cocrystal systems ground for less than 5 min, blue symbols represent cocrystal systems, red symbols represent samples being X-ray amorphous after various grinding times, green symbols represent the amorphous samples after crystallization, forming the cocrystal. (**b**) Loadings plot of PC-1. (**c**) Loadings plot of PC-2.

To gain a deeper insight into the transformation of the physical mixture into the cocrystal during dry-grinding, principal component analysis (PCA) of the Raman spectra was performed. PCA is a valuable technique to visualize differences in the cocrystal formation rate and can help to provide insight into solid-state transformation processes taking place during grinding [15]. In Figure 10a, the PCA 2D score plot of piracetam-citric acid cocrystal formation is presented. Of the total variance, 70% is explained by the first two components. Principal component 1 (PC-1, 50% of the total variance) distinguishes between the cocrystal and non-cocrystal systems: It correlates negatively with non-cocrystal and positively with cocrystal systems. Comparison of the PC-1 loadings plot with the Raman spectra of the physical mixture and the cocrystal supports this interpretation of the score plot (Figure 10b). Scores of samples dry-ground for less than 5 min cluster in the left part of the score plot (black symbols). XRPD confirms that these samples remain as physical mixtures of the original crystalline components. Scores of samples ground for more than 5 min forming cocrystals (confirmed by XRPD) cluster on the right hand side of the score plot (blue symbols). In some cases, the samples are completely X-ray amorphous after different milling periods (red symbols). When these amorphous samples were stored for 24 h at ambient conditions, they crystallized and clustered with the cocrystal scores, indicating cocrystal formation (green symbols). PC-2 (20% of the total variance) describes spectral differences between the physical mixtures of citric acid and piracetam form I or form III (PC-2 loadings plot Figure 10c). After completion of the cocrystal formation, no differences between the spectra of the cocrystals formed by form I and form III are detected with PCA.

**Figure 11.**
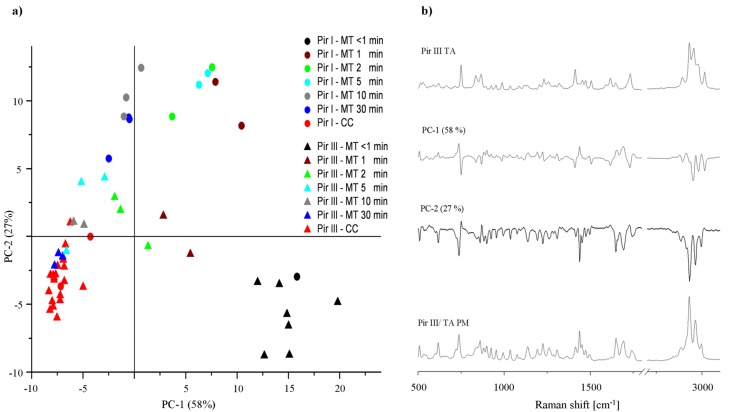
(**a**) PCA 2D score plot of piracetam form I or form III dry-ground with tartaric acid. (**b**) Loadings plots of PC-1 and PC-2, Raman spectra of Pir III/TA PM and Pir III TA for comparison.

In Figure 11a the PCA 2D score plot of piracetam-tartaric acid cocrystal formation is displayed. The first two PCs explain 85% of the total variance: PC-1 with 58% and PC-2 with 27%. PC-1 differentiates between cocrystal and non-cocrystal systems and correlates negatively with cocrystal and positively with non-cocrystal spectral information. This interpretation is confirmed if the PC-1 loadings plot is compared with the Raman spectra of both the physical mixture and the cocrystal (Figure 11b). The differences in PC-2 space are harder to attribute, and are discussed below. Interestingly, the mixtures prepared with form I at less than one minute of milling occupy the same PCA space as those prepared with form III at the same period of milling. XRPD analysis revealed that form I crystallized to form III within this time period. With increasing milling time for form III, the score clusters move from the physical mixture cluster in quadrant IV towards the cocrystal cluster in quadrant III. After 10 min of grinding, the scores cluster with the cocrystal reference scores suggesting completed cocrystallization. Despite form I converting to form III, the mixtures originally consisting of forms I and III take different paths in PCA space and the rate of cocrystal formation is different, with the original form I mixtures taking longer than 30 min to form cocrystals. This suggests some sort of residual structural differences (e.g., in morphology or degree of disorder) of the piracetam crystals in the samples even though both contained form III during the first minute of milling.

XRPD analysis of the samples during milling while diverging in the PC-2 space was also performed. Interestingly, XRPD analysis did not reveal any solid-state differences between the mixtures prepared with the two different polymorphs during the milling process, and the PC-2 loadings of the Raman spectra did not provide evidence that the Raman spectra were the result of different solid-state forms. Therefore, the structural differences associated with this divergence in PCA space are subtle, and may be due to slight differences in the degree of disorder. Nevertheless, the differences in cocrystal formation kinetic are substantial, suggesting that subtle structural differences can have a significant impact on cocrystal formation kinetics.

## 4. Conclusions

Piracetam cocrystals containing citric acid and tartaric acid, respectively, can be prepared by dry-grinding as well as liquid-assisted grinding. The rate of cocrystal formation is independent of the polymorphic form of the API, but differs with the applied grinding technique. As expected, the cocrystal formation during liquid-assisted grinding is faster than that during dry-grinding. Piracetam-citric acid cocrystals are formed via an amorphous intermediate stage. This could not be confirmed for the piracetam-tartaric acid cocrystals. However, subtle structural differences were found to influence the piracetam-tartaric acid cocrystal formation process. This study shows that Raman spectroscopy in combination with principal component analysis is a valuable tool to follow cocrystallization processes during different milling techniques.
